# Examining the condition-specific antisense transcription in *S. cerevisiae* and *S. paradoxus*

**DOI:** 10.1186/1471-2164-15-521

**Published:** 2014-06-25

**Authors:** Krishna B S Swamy, Chih-Hsu Lin, Ming-Ren Yen, Chuen-Yi Wang, Daryi Wang

**Affiliations:** Institute of Molecular Biology, Academia Sinica, Taipei, 115 Taiwan; Biodiversity Research Center, Academia Sinica, Taipei, 115 Taiwan

**Keywords:** Antisense RNA, Evolution, Budding yeast, Stress

## Abstract

**Background:**

Recent studies have demonstrated that antisense transcription is pervasive in budding yeasts and is conserved between *Saccharomyces cerevisiae* and *S. paradoxus.* While studies have examined antisense transcripts of *S. cerevisiae* for inverse expression in stationary phase and stress conditions, there is a lack of comprehensive analysis of the conditional specific evolutionary characteristics of antisense transcription between yeasts. Here we attempt to decipher the evolutionary relationship of antisense transcription of *S. cerevisiae* and *S. paradoxus* cultured in mid log, early stationary phase, and heat shock conditions.

**Results:**

Massively parallel sequencing of sequence strand-specific cDNA library was performed from RNA isolated from *S. cerevisiae* and *S. paradoxus* cells at mid log, stationary phase and heat shock conditions. We performed this analysis using a stringent set of sense ORF transcripts and non-coding antisense transcripts that were expressed in all the three conditions, as well as in both species. We found the divergence of the condition-specific anti-sense transcription levels is higher than that in condition-specific sense transcription levels, suggesting that antisense transcription played a potential role in adapting to different conditions. Furthermore, 43% of sense-antisense pairs demonstrated inverse expression in either stationary phase or heat shock conditions relative to the mid log conditions. In addition, a large part of sense-antisense pairs (67%), which demonstrated inverse expression, were highly conserved between the two species. Our results were also concordant with known functional analyses from previous studies and with the evidence from mechanistic experiments of role of individual genes.

**Conclusions:**

By performing a genome-scale computational analysis, we have tried to evaluate the role of antisense transcription in mediating sense transcription under different environmental conditions across and in two related yeast species. Our findings suggest that antisense regulation could control expression of the corresponding sense transcript via inverse expression under a range of different circumstances.

**Electronic supplementary material:**

The online version of this article (doi:10.1186/1471-2164-15-521) contains supplementary material, which is available to authorized users.

## Background

Recent transcriptomic studies have revealed that genome-wide pervasive transcription is widespread in prokaryotes and eukaryotes [1–5]. The pervasive transcription is comprised of many non-coding RNAs (ncRNAs). Some of these ncRNAs are transcribed from the opposite DNA strand of the protein-coding sequences and overlap in part with the sense RNAs, forming an interspersed transcriptional organization [6, 7]. These are known as antisense RNAs (asRNAs or *cis*-natural antisense transcripts). The role of antisense transcription has been relatively well studied in mammals, plants, and metazoans [8–11]. Studies have also explored the level of conservation of antisense transcripts during vertebrate evolution [12]. In addition, antisense transcription has been identified in diverse yeasts including sensu stricto yeasts, *Kluyveromyces lactis* and *Schizosacharomyces pombe* [8, 13]. Experimental validation of antisense transcription indicates that they can repress sense transcription through transcriptional interference and histone deacetylation [14, 15]. As an example, antisense transcripts have been demonstrated to control genes such as the meiosis regulator gene *IME4* [14], a gene that mediates *MAT* and nutritional control of meiosis [16]. Other examples include the role of antisense transcription in control of *PHO4* [15], the phosphate metabolism gene and *GAL10*, the galactose metabolism gene [17].

Recent studies have independently demonstrated that antisense transcription is evolutionary conserved in bacteria [3], yeast species [18] and mammals [12, 19]. In addition, antisense transcripts are pervasive, exhibit conserved expression levels and localize at the 3′ end of genes. Conserved antisense transcribed regions also have lower sequence divergence compared to the unconserved regions. Moreover, putative antisense transcripts of *S. cerevisiae* show inverse expression relative to the their sense transcripts at stationary phase and stress conditions relative to their expression levels at mid-log phase [8, 20]. They have also been validated for function in relevant conditions. However, the existing studies [8, 18] have either explored the evolutionary conservation of pervasive antisense transcription between *S. cerevisiae* and *S. paradoxus* or have explored the response of antisense transcription in different conditions in an individual species. The evolutionary characteristics of antisense transcription in different conditions are still unclear.

High throughput RNA-sequencing (RNA-seq) has been successfully applied to study transcriptomics with demonstrated advantages over low throughput methodologies such as microarrays, EST, CAGE and SAGE. Polyadenylated (poly A+) sequencing protocol was employed by recent studies to identify antisense transcripts [8, 21]. Goodman et al. [18], on the other hand used ribo-minus RNA sequencing and identified evolutionary conservation of antisense transcription in yeasts cultured at mid-log condition. The ribo-minus sequencing strategy indeed has some advantages over poly A + sequencing method. The key difference between the two strategies comes from the fact that an estimated 70% or more of nuclear RNA are not poly-A tailed. In our study, we employed the conservative poly A + protocol over ribo-minus protocol to perform ultra deep RNA pair-end sequencing, so that a fair comparison could be made with the previous studies, which used the same protocol in different conditions [8]. Furthermore, a comparison of our results with Goodman et al. [18] also would provide a broader view on pervasive antisense transcription in yeast. Our sequencing yielded up to 11 million reads, and thus we believe using poly A + protocol provides adequate information to perform a comparative study in yeasts.

Here, we performed a genome-scale computational analysis to determine the condition-specific evolutionary characteristics of antisense transcription between *S. cerevisiae* and *S. paradoxus* at mid-log phase (ML), early stationary phase (ES) and heat shock (HS) conditions. We used massive parallel sequencing to sequence a strand-specific cDNA library from RNA isolated from *S. cerevisiae* and *S. paradoxus* cells at these three culture conditions. Our findings are largely concordant with some recent studies [8, 14, 15, 17, 18, 22] on evolutionary relationship of the antisense transcription in *S. cerevisiae* and *S. paradoxus* at ML and condition-specific features of antisense transcription in *S. cerevisiae*. The expression levels of conserved antisense transcripts between the two species (*S. cerevisiae* and *S. paradoxus*) under ML, ES, and HS conditions are visibly more divergent than their sense counterparts. This trend remains consistent when antisense and sense transcription levels in the same species are compared across different conditions. This suggests that antisense transcripts could mediate sense regulation more often during changes in environmental conditions.

In addition, several sense-antisense pairs displayed inverse trends demonstrating induced and repressed expression in ES or HS relative to the ML in both *S. cerevisiae* and *S. paradoxus.* Interestingly, a majority of the sense-antisense pairs are conserved between the two species. These findings suggest that antisense mediated regulation might induce or repress sense transcription in cellular pathways according to the cell’s requirements in different environmental conditions. The high levels of conservation of this phenomenon in the two sensu stricto yeast species indicate that inverse expression is one of the plausible modes of antisense mediated regulation. However, besides inverse expression, antisense transcripts can certainly mediate sense regulation by a diverse range of alternative mechanisms that need to be explored in future studies. Given that the understanding of antisense transcription is still limited, our study can provide insight into the evolutionary changes of antisense transcription in related yeast species under general stress conditions. Our predictions about antisense transcription can also be a starting point for experimental screening of regulatory mechanisms governing cell responses in these stress conditions.

## Methods

### Yeast strains, growth conditions and treatments

To study the evolutionary characteristics of antisense transcription with respect to the change in environmental conditions, the strand-specific total RNA was sequenced from the laboratory strain BY4741; a descendant of S288C in *Saccharomyces cerevisiae* and *Saccahromyces paradoxus* strain a-CC154; a derivative of CBS432. (REF: Curtsey Dr. Wen-Hsuing Li). To elucidate the response of antisense RNA-regulated genes to the changes in growth phase and temperature, these two yeast strains were cultured under three different conditions: mid-log phase, early stationary phase and heat-shock.

Cultures were grown in YPED medium (1.5% yeast extract, 1% peptone, 2% dextrose, 2 g/L SC Amino Acid mix, 100 mg/L adenine, 100 mg/L tryptophan, 100 mg/L uracil) at 30°C. The cells were harvested at mid-log phase with the OD600 values being 0.802 (*S. cerevisiae*) and 0.827 (*S. paradoxus*). For early stationary phase, the cells were taken two hours after the glucose levels reached zero, for which glucose levels were monitored hourly using Glucose (HK) assay kit (Sigma-Aldrich, MO, USA). OD600 values for early stationary phase were 2.22 (*S. cerevisiae*) and 2.217 (*S. paradoxus*). The samples were collected by harvesting 12 ml of mid-log and early-stationary cultures and were quenched by adding to 20 ml pre-chilled liquid methanol at a final concentration of 60%, which was later removed by centrifugation, washed in RNase-free water and stored these overnight at -80°C. For heat shock culture, 12 ml of mid-log culture was harvested by centrifugation. Cells were re-suspended in 10 ml of 42°C pre-warmed YPD medium and put in a 42°C water bath for 15 minutes and quenched by adding to 20 ml liquid pre-chilled methanol, which was later removed by centrifugation, washed in RNase-free water and stored these overnight at -80°C.

### Construction of strand-specific cDNA library

The strand-specific cDNA library was constructed by dUTP second-strand method [23], which was identified as the leading protocol for pair-end sequencing [24]. Briefly, incorporation of deoxy-UTP during the second-strand cDNA synthesis and subsequent destruction of the uridine-containing strand in the sequencing library were responsible for identifying the orientation of transcripts.

Total RNA was isolated with Yeast RNA Purification Kit (MasterPure™ Epicentre). Genomic DNA contamination was removed with Turbo DNase (Ambion) followed by phenol chloroform extraction. The cDNA libraries for RNA samples were constructed using TruSeq RNA Sample Preparation Kits v2 based on the guide (Illumina, Cat. No RS-122-2001). PolyA-containing mRNA was purified using 50 μl RNA Purification Beads for each sample. The purified RNA fragments were reverse-transcribed into first-strand cDNA using SuperScript II Reverse Transcriptase (Invitrogen, Cat. No 18064–014) and random primers (Illumina, Cat. No. RS-122-2001). The first strand cDNA/RNA hybrid was precipitated with ethanol and ammonium acetate. The second-strand cDNA synthesis was performed in a 80 μl reaction volume using NEBNext® mRNA Second Strand Synthesis Module (NEB, NEB # E611lL) and dNTP/dUTP mix (Fermentas, Cat. No. R0251, dTTP → dUTP) at 16°C for 2.5 hours. The DNA fragments were purified by QIAquick PCR Purification Kit (Qiagen, Cat. No 28106) and treated with T4 DNA polymerase, E. coli DNA polymerase I Klenow fragment and T4 Polynucleotide Kinase in a 30 μl reaction using the buffer and the enzyme in the kit (Illumina, Cat. No RS-122-2001) at 37°C for 30 min. The blunt phosphorylated DNA fragments were purified by the MinElute PCR Purification Kit (Qiagen, Cat. No 28006) and treated with Klenow fragment of DNA polymerase lacking exonuclease activity in the presence of dATP to add an adenine overhang to the 3′ ends of each strand in a 30 μl reaction using the kit (Illumina, Cat. No. RS-122-2001) at 37°C for 30 min. Adapters required for sequencing on the Illumina platform were added to DNA fragments by ligation in a 37.5 μl reaction using the kit (Illumina, Cat. No. RS-122-2001). The ligation products were purified by the MinElute PCR Purification Kit (Qiagen, Cat. No 28604) and eluted the ligation products in the size range from 350 to 450 bp from the gel. These products were precipitated and resuspended in 21 μl TrueSeq Resuspension Buffer using QIAquick Gel extraction Kit (Qiagen, Cat. No 28704) and treated with USER™ Enzyme mixture (Uracil-Specific Excision Reagent, NEB, Cat. No M5505L) to degrade the dUTP in the coding strand (second strand). The adapter-modified DNA fragments were amplified by the kit (Illumina, Cat. No. RS-122-2001). The amplified products were purified by Agencourt AMPure XP (Beckman, Cat. No A63881) and collected in 30 μl Resuspension Buffer (Qiagen, Cat. No 28604). After quantification by Quant-iT dsDNA HS Assay Kit (Invitrogen, Cat. No Q32851) and KAPA QPCR Library Quantification Kit (KAPABiosystem. Cat. No kk4822), calculation of molar concentration and quality examination by Expersion DNA 1 K Analysis Kit (Bio-Rad Cat. No 700–7107), this DNA library was prepared for sequencing.

### Illumina sequencing

RNA pair-end sequencing was performed by Illumina Hiseq2000 (San Diego, CA, USA) with standard protocol. Amplified material was loaded onto a flow-cell at a concentration of 10 pM. Sequencing was carried out on the Illumina Hiseq2000 by running 100 cycles according to the manufacturer’s instructions. The image convolution and calculation of quality value were performed using Goat module of Illumina pipeline v.1.8. Sequenced reads were generated by base calling using the Illumina standard pipeline. For each sample, our sequencing yielded around 11.6 million 101-nucleotide paired-end reads (range from 10.95 million to 12.12 million).

### Identification of transcripts in *S. cerevisiae*and *S. paradoxus*

We used Bowtie [25] and TopHat [26] to map the 101-nucleotide paired-end reads to the genome. Reads were annotated based on the known genome sequences and annotations from Saccharomyces Genome Database (http://www.yeastgenome.org) for *S. cerevisiae* and from the study of Liti et al. [27] for *S. paradoxus*. The sequencing reads that mapped to the unique position on the genome were further selected from total mapped reads for further analysis (~92% of total mapped reads).

Furthermore, to determine the complete transcriptional landscape, we designed a method to detect all the transcripts. The read depth (i.e., the number of reads at a given position) was used to define a transcript unit and the transcript boundaries were manually annotated. For the computational detection of each transcript unit, we iterated the following four-step process. *(i)* We selected a position with the highest read depth as the starting point. We also used the criteria that for any position to be considered in this analysis, it should have a read depth greater than or equal to five. *(ii)* We extended the boundary in both directions (upstream and down stream) by one base pair until the mean read depth of the transcript was at least four times higher than the read depth of the neighboring position. *(iii)* The boundary of this transcript was reassigned to include the reads that contained the position at which the mean read depth satisfied the criteria in step *(ii). (iv)* The region that was already assigned as a transcript was flagged and excluded from the following cycles. In addition, the BPKM (bases per kilo-base of gene model per million mapped bases) was calculated [28] for each annotated transcripts for different samples and used as a measure of expression level. The observed transcripts were classified as ORF transcripts (ORF-Ts) if they overlapped with verified and uncharacterized ORF boundaries in the same orientation. A transcript was assigned as a non-coding RNA (ncRNA) when there was no overlap with known verified and uncharacterized ORF in the currently available genome annotation. When an ORF-T and an ncRNA had different orientation and overlapped by at least 1 bp, they were coupled as a pair of sense-antisense pair and the ncRNA was assigned as an antisense transcript.

### Estimation of sense and antisense expression over a canonical gene model

In order to understand the expression landscape over a canonical gene model, we computed the expression densities of sense and antisense transcripts based on their BPKM values. Prior to the density calculation, the expression BPKM value of each transcript position was normalized by the mean of sense and antisense BPKM values. The region between transcription start site (TSS) and transcription termination site (TTS) of each canonical gene model was divided into 200 equal intervals [18] *.* The normalized BPKM values of these intervals were used as an estimate of expression density of a gene. However, for regions 100 nucleotides upstream and down stream of TSS and TTS respectively, the coverage was represented directly by adapting the method used in Goodman et al. [18] *.* We then calculated the mean densities of all transcripts.

### Estimation of expression variation

In order to understand the relationship between expression level and expression conservation, we determined the conservation of sense-antisense pairs, by comparing the variation of expression between the two species *S. cerevisiae* and *S. paradoxus* in mid-log condition or other culture condition. For each sense and antisense transcripts, the expression variation was computed from the absolute value of logarithmic expression difference between *S. cerevisiae* and *S. paradoxus*.

### Estimation of sense-antisense pair conservation

**Figure 1:**
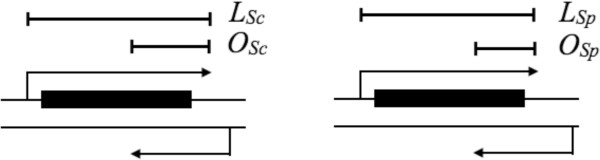
**Toy diagram for the estimation of conservation score.** Here *L*_*i*_ and *O*_*i*_, where *i* = *Sc* or *Sp* are the total length of the sense region and length of the overlapping region between sense and antisense transcripts respectively. In this ORF, conservation score is computed by employing the fraction of region *O*_*sp*._

Next, to determine the level of conservation of sense-antisense pairs between *S. cerevisiae* and *S. paradoxus*, we calculated the fraction of regions that formed sense-antisense pairs and were conserved between the two species. For each ORF transcript, a conservation score was computed as the minimum fraction of sense-antisense pairs conserved between the two species divided by the mean of the two species. This can be represented by the equation below and Figure 1 contains the corresponding toy diagram.


where, *S* denotes the conservation score, which indicates the conserved fraction of regions forming the sense-antisense pair in both the species; *L*_*i*_ is the total length of the sense region and *O*_*i*_ is the length of the overlapping region between sense and antisense transcripts where *i* = *S. cereviaise (Sc)* and *S. paradoxus (Sp)*; *C*_*Sc*_ and *C*_*Sp*_ is the percentage or fraction of the sense transcript overlapped by the corresponding antisense transcript in *S. cereviaise* and *S. paradoxus* respectively. Here, we would like to point out that the exact sequence identity between the species was not considered while arriving at conservation score. The conservation score thus represents the length of overlap between sense and antisense transcripts found in both the species. In other words, if lengths of overlapping fraction *C*_*sc*_ and *C*_*Sp*_ in the two species are very similar then conservation score *S* will be close to 1 and if *CSc* and *CSp* are disparate, *S* will be close to 0.

## Results

To evaluate the conservation of antisense transcripts in yeast, we constructed stranded-specific cDNA libraries of two yeast species, *S. cerevisiae* (*Sc*) and *S. paradoxus* (*Sp*) under three different culture conditions, including mid-log phase (ML), early stationary phase (ES), and after heat shock (HS). We used the dUTP method to retain the strand specificity [23], and followed this with ultra-deep sequencing (Illumina) to sequence the cDNA libraries. Our sequencing yielded 10.7 to 11.4 million of unique paired-end reads for each sample. We analyzed all the reads that map the unique position in genomes and found 7,254 and 7,248 transcript units in *Sc* and *Sp*, respectively (Table 1). Although the total number of transcripts was similar between the two species, the number of non-coding transcripts in *Sc* was less than in *Sp*. We identified a total of 5,472 ORF-T units and 2,403 ncRNA units (ORF-T and ncRNA units defined in Material and Methods).

**Table 1:** **Overview of transcript units data from** ***S. cerevisiae*** **(** ***Sc*** **) and** ***S. paradoxus*** **(** ***Sp*** **)**

	ORF-T	ncRNA	Others	Total transcripts
Present in both *Sc* and *Sp*	5159	1057	167	6383
Unique to *Sc*	285	528	58	871
Unique to *Sp*	28	818	49	895
Total	5472	2403	274	8149

On closer inspection, we found 5,159 ORF-Ts (95%) present in both species, 285 were present only in *Sc*, and 28 were unique to *Sp*. For the 2,403 ncRNA units, 1057 were conserved in both species (43%), 528 were only found in *Sc* and 818 were unique to *Sp* (Table 1). This suggests that overall the pattern of sense transcription, which is derived from ORF-Ts is similar in *Sc* and *Sp*. Nevertheless, a visible difference in ncRNA units that are unique to the each species is seen and suggests the possibility that antisense transcription in the two species, which is derived from ncRNA units, could be expected to be relatively more divergent between species than sense transcription. However, the ncRNA units unique to *Sp* may contain false positives due to the incomplete annotation of ORFs in *Sp*. To further understand the function and divergence of antisense transcripts and to overcome the ambiguity due to disparity in ncRNA units between the two species, we selected a subset of *Sc* ORF-Ts, which had homologous ORF-Ts in *Sp* and also overlapped with antisense transcripts. Our selected data set constituted of 1,053 ORF-Ts and 956 ncRNAs.

### Assessing the distribution of sense and antisense transcription levels

**Figure 2:**
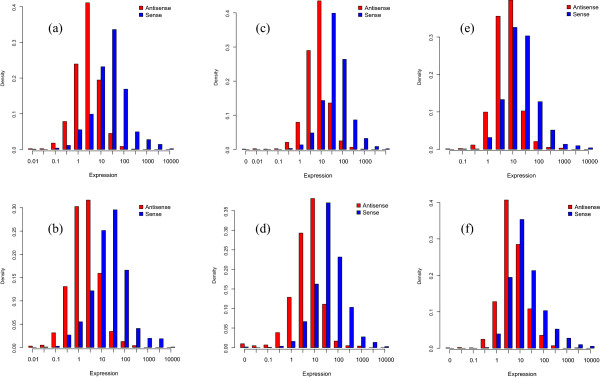
**Histogram of sense and antisense transcription levels in** ***Sc*** **and** ***Sp*** **from the three culture conditions. (a)** *Sc*-ML, **(b)** *Sp*-ML, **(c)** *Sc*-ES, **(d)** *Sp*-ES, **(e)** *Sc*-HS, and **(f)** *Sp*-HS conditions. (See Methods for details on Density and Expression).

As expected, the number of sense transcripts was found to be more abundant than their antisense counterparts in all the three culture conditions and they corroborated some of the findings of recent studies [8, 18]; albeit with a few minor differences in the appearance and location of maximum when compared to Goodman et al. [18]. The distribution of sense transcription levels in ML in both *Sc* and *Sp* are right shifted when compared to the antisense transcription levels, indicating the higher abundance of sense transcripts than the antisense transcripts (Figure 2a and b). Though the distribution around the maximum BPKM value (expression) is more condensed, it is largely similar to the findings of Goodman et al. [18]. This difference may be attributed to difference in the sense and antisense reference gene models in the two studies. In our model, we annotated the antisense transcript boundary and quantified the reads in annotated antisense regions (See Methods). While the study by Goodman et al., quantified the antisense transcript by only considering the overlap of antisense sequence reads in known ORFs. Moreover, since our dataset only includes genes with antisense expression, our results start from non-zero expression value (derived from BPKM for each gene). Furthermore, similar to the previous studies by Goodman et al. [18] and Yassour et al. [8], we also found that a majority of sense transcripts (>80%) overlapped with known gene annotations, while only a small fraction (10%) of antisense transcripts could be assigned to previously known gene annotations.

As it was observed by Goodman et al. [18], the antisense transcript usually had a lower expression and only overlapped a fraction of the sense transcript. Also, a comparison of the combined pattern of antisense transcription to the sense transcription suggested that the antisense transcription is most likely to occur from the 3′ end of the genes (results not shown). This feature was consistent in all the three culture conditions examined in our study. To verify whether the frequent initiation of antisense transcript at 3′ end was related to the paucity of introns in *Sc*, we downloaded the intron containing genes from Ares lab Yeast Intron Database (Version 3.0) [29]. We found that a total of 249 ORFs in *Sc* had introns and 47 (18%) of these ORFs formed sense antisense pairs. Among these while only 3/47 pairs had antisense transcript arising from the intron region, the rest were initiated outside the intron region and from 3′ end of the gene. In addition, we found that the frequency of sense-antisense pairs (18%) in intron containing ORFs was only slightly less than the frequency of sense-antisense pairs (24%) in the whole genome of *Sc.* A recent study in *Sc* [30] demonstrated that initiation of antisense transcription at 3′ end of an ORF was associated with the presence of a pre-initiation complex (PIC). They showed that on insertion of short 3′ end regions in the middle of other genes; antisense transcripts were initiated and such antisense transcripts could suppress sense transcripts.

Introns are common in genomes of vertebrates. We compiled the available data on antisense transcription from published human and mouse studies to configure their relationship with introns in vertebrates. [31–33]. The frequency of sense-antisense pairs in intron-containing genes in human and mouse was high, where close to 50% of sense-antisense pairs were found in intron containing genes and several of these pairs actually arose from intron regions. However, a study in human and mouse by Sun et al. [34] showed that putative sense-antisense pairs overlapping at the 3′-UTRs are significantly more frequent than those overlapping at their 5′-UTRs, suggesting preferential targeting of 3′-UTRs by antisense transcripts. Taken together, these results suggest that antisense transcripts are more likely to have a preference to initiate from 3′ end, than being biased by the lack of introns in *Sc*.

Figure 2c to f illustrate the distributions of transcription levels of sense and antisense transcripts in ES and HS. In the case of ES and HS, the distributions of antisense transcripts are progressively more right shifted than that of ML in *Sc* and *Sp* (Wilcoxon Rank-Sum test *p*-value < 0.001), suggestive of more abundant antisense transcripts in the environmental stress response conditions. To validate this possibility we examined the sense-antisense interactions in ES and HS by using sense-antisense pairs present in both the species. We found 538 sense-antisense pairs (43%) with the sense transcription fold change < 1 (i.e.*,* repressed) and antisense transcription level fold change > 1 (i.e., induced) in either ES or HS relative to ML. This data constituted 102 pairs in ES, 284 in HS and 152 in both that were induced in antisense. We also found 189 pairs where the sense was induced, while the antisense was repressed (75 in ES, 85 in HS, 29 in both). Several of these pairs with induced sense transcripts and repressed antisense transcripts showed similar response to HS and ES, suggesting the possibility that they might be involved in facilitating the adaptation of metabolic processes in species during stress [8, 35].

On examining the functional annotations of these sense-antisense pairs, we found the pairs with induced sense and repressed antisense in ES relative to ML were important in the carbohydrate metabolic process, mitochondrial function, respiration, and starvation response (for example, *GAL4*, *PAM16*, *PET10*, *ARF2*) consistent with the data of Yassour et al. [8]. On the other hand, genes, with repressed sense and induced antisense from ML to ES, were enriched in glycolysis and fermentation (for example, *GPM1* and *ADH6*), in agreement with Yassour et al. [8]. Furthermore, some sense genes known to be important under environmental stresses were induced while the antisense was repressed (for example, *CRF1*, *MRK1*, *BDF2*, and *PAU2*) [8]. In comparison with the findings of Yassour et al. [8], we found 32 pairs in common. Among the remaining 28 pairs present in Yassour et al. [8] but not in our data set, five pairs did not demonstrate sense expression and hence were excluded from the analysis, 17 pairs lacked antisense expression and six pairs did not overlap with ORF-T transcripts. Among the pairs that lacked antisense expression, seven pairs were common with a previous study [36], suggesting that our results to be robust.

### Comparison of sense and antisense transcription across species and culture conditions

**Figure 3:**
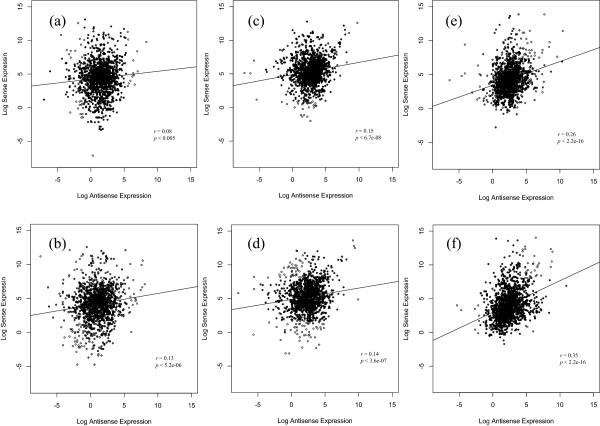
**The correlation of expression levels between sense and antisense transcription in ML, ES and HS conditions in** ***Sc*** **and** ***Sp.*** Expression is derived from logarithm of sense and antisense BPKM values from our selected dataset. For both species the ES and HS conditions have slight positive correlation between antisense and sense transcript levels, which is not evident in *Sc*-ML condition. Here **(a)** *Sc*-ML, **(b)** *Sp*-ML, **(c)** *Sc*-ES, **(d)** *Sp*-ES, **(e)** *Sc*-HS, and **(f)** *Sp*-HS conditions.

In concordance with the previous studies [8, 18, 37], the correlation in ML condition in *Sc* (Figure 3a) suggested that antisense transcription is not directly coupled with transcription activity of the sense strand. However, in ES and HS conditions, we found that the antisense transcription activity is slightly positively correlated with the sense transcription (Figure 3d and f). In the case of *Sp*, we found that sense and antisense are positively correlated in all three conditions. On closer inspection, we found that for both species in HS, correlation was 0.35 and 0.26 with *p*-value < 2.2 × 10^-16^, which was considerably higher than ML and ES. The higher number of pairs with induced and repressed antisense expression in HS (285 and 85) than in ES (102 and 75) also corroborates with higher correlation levels in HS. Taken together, these results suggest the possibility that the interplay of sense-antisense transcription is likely to be more prominent in HS than in other conditions.

Next, to assess the relationship between sense and antisense transcription levels, we examined their correlation between *Sc* and *Sp* in the three culture conditions (Table 2). The general trend in Table 2 suggested that sense transcription is less divergent when compared to antisense in the three culture conditions when we consider both “*the same species and in different conditions*” and “*different species and in same conditions*”. For example, from Table 2, “*the different species and in same conditions*” for sense transcription (*SpSc*-ML: 0.88 > *SpSc*-ES: 0.84 or > *SpSc*-HS: 0.82) are more correlated than that for antisense (*SpSc*-ML 0.64 > *SpSc*-ES: 0.64 or > *SpSc*-HS: 0.56), where *SpSc* stands for correlation between *S. cerevisiae* and *S. paradoxus*. While, the “same species in different condition”, denotes relationship between sense and antisense transcription within a species under different conditions; the “different species in the same condition” can tell us about this relationship across species. The observed difference could be either due to a species-specific effect, condition-specific effect or a combination of both. However, on considering the conglomerate of the level of divergence of sense and antisense transcription from combinations of species and conditions, we can speculate that the condition-specific antisense transcription levels has higher divergence compared to condition-specific sense transcription levels. These observations suggest the potential role of antisense transcription in response to the change from ML to ES or HS and could play an important role in facilitating the processes by which yeasts adapt to change in environmental conditions.

**Table 2:** **Correlation of expression levels across species and culture conditions**

Sense expression correlation						
	***Sc***-ML	***Sp***-ML	***Sc***-ES	***Sp***-ES	***Sc***-HS	***Sp***-HS
*Sc*-ML	**1**	0.88	0.57	0.48	0.53	0.57
*Sp*-ML	0.64	**1**	0.45	0.47	0.42	0.56
*Sc*-ES	0.61	0.32	**1**	0.84	0.58	0.52
*Sp*-ES	0.41	0.47	0.64	**1**	0.45	0.45
*Sc*-HS	0.49	0.23	0.39	0.17	**1**	0.82
*Sp*-HS	0.31	0.40	0.09	0.15	0.56	**1**
Antisense expression correlation						

The values on the top (and bottom) of the negative diagonal elements (indicated in bold) are the correlation values of sense transcript (and antisense transcript) in *S. cerevisiae* (*Sc*) and *S. paradoxus* (*Sp*) respectively. ML, ES and HS correspond to the Mid-Log, Early stationary phase and Heat-shock conditions. The horizontal and vertical column has *species name*-experimental conditions used for computing correlation values.

**Figure 4:**
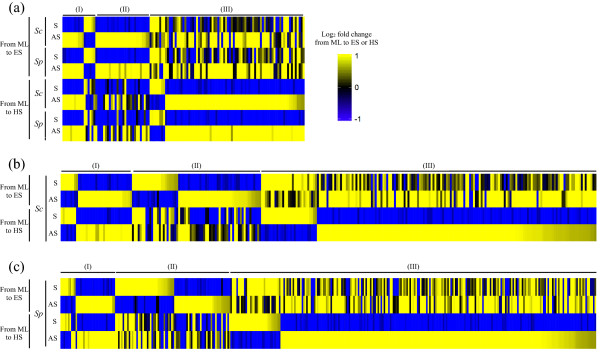
**Significant inverse expression patterns of sense-antisense pairs conserved in** ***Sc*** **and** ***Sp*** **. (a)** Sense-antisense pairs that show inverse expression patterns conserved in *Sc* and *Sp* from ML to (I) both ES and HS, (II) only ES, (III) only HS. **(b&c)** Sense-antisense pairs that show inverse expression patterns only in **(b)** *Sc* or **(c)** *Sp* from ML to (I) both ES and HS, (II) only ES, (III) only HS.

Furthermore, we wanted to investigate the patterns of change in sense and antisense transcription in different conditions, by considering both species specific and conserved sense-antisense pairs. For this, we examined sense and antisense transcripts that show inverse trends and change significantly from ML to ES or HS in both *Sc* and *Sp* in terms of their BPKM ratios. We selected sense-antisense pairs with inverse expression patterns and had at least a 1.5 fold change [8, 37, 38] from ML to ES or HS in *Sc* and *Sp*. We found that some of these genes (Figure 4 and Additional file 1: Table S1) had significant changes from ML to HS in *Sc* only, while several had this feature conserved between *Sc* and *Sp*. As shown in Table 3, 328 pairs had repressed sense and induced antisense expression patterns in either ES or HS relative to ML (68 in ES, 209 in HS, 51 in both) only in *Sc*. Similarly, 385 pairs demonstrated this feature only in *Sp* (49 in ES, 277 in HS, 45 in both). Interestingly, for 92 sense-antisense pairs this pattern was conserved in both species (20 in ES, 61 in HS, and 11 in both). On the other hand, there were 78 pairs with induced sense and repressed antisense (31 in ES, 37 in HS, 10 in both) in *Sc* only and 99 pairs in *Sp* only (50 in ES, 41 in HS, 8 in both) and 13 pairs were conserved in both species (5 in ES, 8 in HS). Taken together, these results suggest that an inverse expression between sense and antisense pairs could mediate regulation of condition-specific sense transcription in either *Sc* or *Sp* and sometimes in both the species.

**Table 3:** **Characteristics of sense-antisense pairs in the two sensu stricto yeasts**

	***S. cerevisiae***	***S. paradoxus***	***Both***
**I-sense, R-antisense**	328	385	92
**R-sense, I-antisense**	78	99	13

I-sense/I-antisense: induced sense/antisense transcription (fold change >1.5), R-sense/R-antisense: repressed sense/antisense transcription (fold change > 1.5).

To validate our results, we compared it with the available experimentally determined sense-antisense relationships. This model was supported by mechanistic studies on *IME4* [14], *PHO84* [15], *KCS1* [39] and *GAL10* [17]. We also found that, the antisense transcripts of *IME4* and *GAL10* expressed and sense transcripts were repressed consistently in all three conditions and in both the species. However, we did not detect the antisense transcripts of *KCS1* that was induced in low phosphate condition [39]. This can be expected, because our study does not include the meiosis phase, low phosphate condition, or galactose treatment. Similarly, we also detected the sense transcript of *PHO84* was repressed and it’s antisense transcript induced from ML to ES, as observed previously in the aging cells [15]. However, this phenomenon was not conserved between *Sc* and *Sp*. Even though the antisense transcript was expressed in both the species, the antisense expression did not satisfy the criterion of a 1.5 fold change. A plausible reason for the lack of conservation could be the difference in domain architecture of *PHO84* between *Sc* and *Sp* [40, 41]. While two general substrate transporter domains of *PHO84* are conserved between the two species, two additional domains with sugar transporter activity are specific to *Sc* and absent in *Sp* [40, 41]. This might cause difference in functionality of antisense transcription between the two species. It suggests a rapid change in antisense regulation and the lineage-specific role of antisense transcript of *PHO84*. In the case of *FLO1*, the antisense expression level was visibly higher in ES (16.7 BPKM) than the ML (1.2 BPKM). Though the role of sense *FLO1* in cell wall binding is previously established, the up-regulation of *FLO1* antisense transcripts during ES is intriguing and its function is unavailable from previous experiments.

Some examples of genes with inverse expression patterns and significant change from ML to ES and HS were conserved in the two species (Additional file 1: Table S1), suggesting its functional importance. For example, ribosome structure protein (*RPP1B*, *RPS19A*, *RPL36B*) and biogenesis protein (*DIM1*) related to translation were repressed in ES and HS. *NUP53* and *NIS1*, which are regulators for mitosis [42, 43] were repressed in HS. *CSG2*, whose function is related to DNA damage, lactic acid, and acetaldehyde stresses [44–46] was induced in ES. *ARF2*, whose null mutant results in the decrease of survival rate in the stationary phase [47] and was also induced in ES. *MRK1*, was known to respond to stress [48] and was induced in HS. *CRF1*, involved in repression of ribosomal protein gene expression in stress and poor nutrients [49], was also induced in HS. *MBR1*, which was related to aerobic fermentation [50], was induced in HS. These findings suggest that antisense transcription might be important in maintaining these cellular functions.

### A large fraction of conserved sense-antisense pair demonstrate inverse expression patterns

It has been hypothesized that antisense transcript mediated regulation function through RNA interactions [6]. To verify the extent of conservation of sense-antisense pairs between *Sc* and *Sp*, with different modes of regulation in the HS and ES relative to ML, we examined the sense-antisense pairs, which demonstrated inverted expression patterns. For this, we arrived at a conservation score: an index of the degree of conservation (Detailed in Materials and Methods). We employed a stringent criteria of conservation score greater than 0.8, i.e., at least 80% of sense-antisense pairs is conserved between *Sc* and *Sp*.

A total of 72 (i.e., 67%) sense-antisense pairs with inverse expression patterns were conserved between the two species under this stringent criterion, supporting the hypothesis. Furthermore, 22 (i.e., 30%) of these conserved sense-antisense pairs demonstrated inverse patterns in ES relative to ML and 55 (i.e., 47%) such pairs were found in HS relative to ML respectively. Whereas, 74 sense-antisense pairs in ES and 118 pairs in HS demonstrated inverse expression but were not conserved between the two species. Next, we determined the distribution of all sense-antisense pairs (independently in ES and ML) with less than 50% of conservation, equivalent to background distribution in both ES and HS conditions. In this background set, we found 15 pairs in ES and 25 pairs in HS had inverse expression relative to ML respectively and were also conserved. On the other hand 21 pairs in ES and 95 pairs in HS demonstrated inverse expression but were not conserved between *Sc* and *Sp* in the background set. Fisher’s exact test was performed between the conserved and not-conserved sense-antisense pairs under stringent criteria and background separately in ES and HS. The *p*-value < 0.05 in both ES and HS suggested that the distribution of conserved stringent sense-antisense pairs was above random expectation. In addition, the proportion of sense-antisense pairs that are conserved under our stringent criterion and demonstrated inverse expression in HS was higher than the pairs in ES according to one-sided two-sample proportion test (*p*-value = 8.61 × 10^-2^). These results suggest that antisense transcript mediated regulation can be expected to be more prevalent in environmental stress such as HS than in starvation stress.

Yeast cells enter a stationary phase when they exhaust available nutrients and are characterized by cell cycle arrest and specific physiological, biochemical, and morphological changes. Some of these changes include thickening of the cell wall and acquisition of thermotolerance [51]. Similarly, for cells under HS, a reprogramming of gene expression, acquisition of thermotolerance, and a transient cell cycle arrest at G_1_ is observed [51–53]. It can thus be speculated that specific sense-antisense pairs are required for maintenance of important cellular function in quiescent and thermal stress induced cells. They might be involved in reprogramming of a transcriptional network to overcome the external stress by inducing or repressing specific pathways. More importantly, the high levels of evolutionary conservation of such sense-antisense pairs, demonstrate the functional importance in related sensu stricto yeasts.

## Discussion and conclusions

By performing massive parallel sequencing of strand-specific cDNA library from RNA of *Sc* and *Sp* cells, which were cultured in three different conditions (i.e., ML, HS and ES), we have attempted to identify the evolutionary characteristics of antisense transcription in sensu stricto yeasts. Overall, the pattern of antisense transcription was similar in both *Sc* and *Sp,* with a lower expression level than sense transcripts. These occurred from the 3′ end of the genes and mostly had a partial overlap with the sense transcript.

Our findings were concordant with previous studies [6, 8, 18] on antisense transcription in yeasts and they also highlighted the importance of antisense regulation in mediating condition-specific transcription in yeasts. A comparison of antisense transcript distribution between the normal ML and the stress inducing HS and ES conditions showed a right shifted distribution in stress conditions, suggesting the key role of antisense transcription in response to change in environmental conditions. Moreover, the inverse expression pattern of sense-antisense pairs in HS and ES relative to ML suggested metabolic changes via regulation when cells are under stress. The functional annotations of our results were largely consistent with a previous study [8]. Furthermore, the varied levels of correlation of sense and antisense expression levels across species and culture conditions (Table 2) suggested that their mechanism of action might be diverse.

Because natural selection preserves functional elements and processes, we know that conservation is an effective method of discerning functional and non-functional cellular processes. Since transcription is a costly process, it is can be assumed that antisense transcription is functional when it is conserved in related species. Previous studies have illustrated the conservation of antisense transcription separately in bacteria [3], yeasts [18] and mammals [12, 19]. These findings suggest that antisense transcription is an evolutionary conserved phenomenon. Although a handful studies have proposed that antisense transcription could be important in stress related condition [8, 36, 37], it remains unclear as to how they mediate regulation with changing conditions and also how conserved is antisense mediated regulation across species.

By examining the antisense transcription in *Sc* and *Sp* cultured in three different conditions, we investigated the evolutionary consequence of antisense transcription with changing conditions. A significantly large fraction of sense-antisense pairs with inverse expression patterns in HS and ES relative to the ML were conserved in both species even when a stringent criterion was imposed. This indicated the possibility that inverse expression could be a plausible way with which antisense mediated sense regulation occurs across yeast species. There could be a dual mode of action of such sense-antisense pairs in yeasts [37]. In addition to reprogramming gene expression pertinent to the changing conditions, it could also lead to greater expression variability for antisense-containing genes, which in turn can help species to adapt to new environments in general. A general examination of, the varied levels of correlation between species and culture conditions (Table 2), together with the high conservation of inverse expression of sense-antisense transcript, highlights the following observation: sense and antisense expression associate in different ways according to the mechanism specified by environmental condition. Some may require a concomitant presence of sense and antisense transcripts, while others work by reciprocated exclusion [22]. Furthermore, such relationships have also been previously observed in mammals [54], suggesting a commonality in the basic mechanism with which antisense regulation works. Furthermore, these results also validate that sense-antisense pairs, which are coupled to serve the different regulatory mechanisms, are conserved in closely related species, in our case *Sc* and *Sp.* Even though our analysis can provide a general understanding of sense and antisense transcription, further experiments will be needed to substantiate the functional characteristics in yeast. There is also a considerable void in experiments testing these mechanistic hypotheses and further research is needed to elucidate these possibilities.

### Availability of supporting data

The RNASeq data set supporting the results of this article is available in the Gene Expression Omnibus (GEO) repository, [http://www.ncbi.nlm.nih.gov/geo/query/acc.cgi?acc=GSE58319].

## Electronic supplementary material

Additional file 1: **Sense-antisense pairs with inverse expression patterns.** (XLSX 106 KB)

## Abbreviations

*Sc*: *Saccharomyces cerevisiae*
*Sp*: *Saccharomyces paradoxus*
ML: Mid-log phase
ES: Early stationary phase
HS: Heat shock condition.
